# From evidence to practice: development of web-based Dutch lipid reference values

**DOI:** 10.1007/s12471-021-01562-x

**Published:** 2021-04-12

**Authors:** N. S. Nurmohamed, D. Collard, J. W. Balder, J. A. Kuivenhoven, E. S. G. Stroes, L. F. Reeskamp

**Affiliations:** 1 Department of Vascular Medicine, Amsterdam University Medical Centers, location AMC, University of Amsterdam, Amsterdam, The Netherlands; 2 Department of Cardiology, Amsterdam University Medical Centers, location VUMC, Vrije Universiteit Amsterdam, Amsterdam, The Netherlands; 3 Department of Paediatrics, University Medical Center Groningen, University of Groningen, Groningen, The Netherlands

**Keywords:** Lipids, Cholesterol, Reference values, LDL‑C, CVD, Prevention

## Abstract

**Introduction:**

In the Netherlands, the total number of yearly measured lipid profiles exceeds 500,000. While lipid values are strongly affected by age and sex, until recently, no up-to-date age- and sex-specific lipid reference values were available. We describe the translation of big-cohort lipid data into accessible reference values, which can be easily incorporated in daily clinical practice.

**Methods:**

Lipid values (total cholesterol, LDL cholesterol, HDL cholesterol and triglycerides) from all healthy adults and children in the LifeLines cohort were used to generate age- and sex-specific percentiles. A combination of RStudio, Cascading Style Sheets and HyperText Markup Language was used to interactively display the percentiles in a responsive web layout.

**Results:**

After exclusion of subjects reporting cardiovascular disease or lipid-lowering therapy at baseline, 141,611 subjects were included. On the website, input fields were created for age, sex and all main plasma lipids. Upon input of these values, corresponding percentiles are calculated, and output is displayed in a table and an interactive graph for each lipid. The website has been made available in both Dutch and English and can be accessed at www.lipidtools.com.

**Conclusion:**

We constructed the first searchable, national lipid reference value tool with graphical display in the Netherlands to use in screening for dyslipidaemias and to reduce the underuse of lipid-lowering therapy in Dutch primary prevention. This study illustrates that data collected in big-cohort studies can be made easily accessible with modern digital techniques and preludes the digital health revolution yet to come.

## What’s new?

- Lipid values are sex- and age-specific and thus require knowledge of normal value ranges for appropriate clinical care.
- We developed the first searchable, national lipid reference value tool with graphical display in the Netherlands.
- This tool can educate both physicians and patients on the normality or abnormality of lipid levels in daily cardiovascular practice and contribute towards prevention of cardiovascular disease.
- This study showed that cohort studies can be used to directly address clinical issues with the use of modern digital techniques and preludes the digital health revolution yet to come.

## Introduction

One of the most important established, causal risk factors for cardiovascular disease (CVD) is dyslipidaemia. In particular, hypercholesterolaemia—which is characterised by elevated low-density lipoprotein (LDL) cholesterol plasma levels—is causally linked to (premature) development of atherosclerosis and subsequent CVD [1]. Plasma lipid levels are routinely and frequently measured in patients with established CVD or a high CVD risk in, for example, the fields of cardiology, neurology, internal medicine and primary medicine. In Dutch primary care alone, the estimated number of yearly measured lipid profiles comprising valuable information on total cholesterol, LDL cholesterol, high-density lipoprotein (HDL) cholesterol and triglycerides currently exceeds 500,000 (data from personal communication).

The interpretation of these measured lipid values can be challenging and requires insight into reference values in the general population. Similarly to other laboratory measurements, lipid values are strongly affected by age and sex [2, 3]. Patient-specific reference values are not only needed for diagnostic decision-making (e.g. genetic testing for familial hypercholesterolaemia), but are also important for creating awareness among physicians and patients of abnormal lipid levels and overcoming the evidence-practice gap in the prescription of lipid-lowering therapies [4]. This gap between patients qualifying for lipid-lowering therapies and those receiving it, is illustrated by the fact that 77% of primary prevention patients and 31% of secondary prevention patients do not receive lipid-lowering therapy despite recommendation to do so in Dutch guidelines on cardiovascular risk management [5].

These and other practical healthcare-related difficulties may be overcome by using data that are generated in big-cohort studies. This type of cohort has been used to study the aetiology, incidence and prognosis of diseases [6], and also, for example, to determine the distribution of lipid levels among the Dutch population [2, 3]. While study outcomes are published in peer-reviewed scientific journals and implemented in guidelines, the actual improvement of clinical decision-making and patient education with this data is lagging. Only a few applications have established routine use in the consulting room, such as U‑Prevent [7]. However, recent technical advances have not only enabled the generation and analysis of ever-increasing datasets [8], but also the development of easy-to-use, graphically attractive and clinically meaningful ways to use research results in actual clinical practice [9].

This report describes the development of an interactive tool based on data from a big Dutch cohort study that provided age- and sex-specific lipid reference values, in an effort to aid in diagnosis of dyslipidaemias and to address the evidence-practice gap in primary prevention.

## Methods

### Study population

For the current analysis, we used data from healthy adults and children in the LifeLines cohort [10], which were extracted from two studies investigating the distribution of lipid levels among the Dutch population [2, 3]. The entire three-generational cohort consists of a representative sample comprising 167,729 persons from the northern part of the Netherlands. Individuals aged between 25 and 50 years were recruited by general practitioners, after which their partner, parents and children were also invited to participate. Adult individuals could also opt to participate through self-registration, after which their family members were invited to participate. All participating subjects signed for informed consent.

Subjects under the age of 8 years old or with a self-reported history of CVD, defined as myocardial infarction, percutaneous coronary intervention, coronary artery bypass grafting or stroke, were excluded. In addition, participants reporting use of lipid-lowering therapies (statins, ezetimibe or fibrates) and non-fasting participants were excluded from the cohort used for the tool development.

Venous blood samples of the remaining participants were collected after an overnight fast. The laboratory procedures for measurement of total cholesterol, LDL cholesterol, HDL cholesterol and triglycerides have been described elsewhere [2].

### Percentile calculation

Percentiles of lipid values of the four lipid classes were made for 5‑year age groups from 20 years and older, after stratification by sex. Children (< 18 years old) and participants aged 18 or 19 years were included as two additional age groups, stratified by sex. For each age and sex group, percentile scores were obtained by ordering the measured lipid values and subsequently assigning the relative ranking in percent rounded to the nearest half of the measured value. We repeated this procedure for every lipid category (total cholesterol, LDL cholesterol, HDL cholesterol and triglycerides).

### Tool development

To interactively display these values, RStudio version 1.2.5019 and R version 3.6.1 were used after installation of the packages Shiny, Dplyr and Plotly (RStudio Inc., Boston, MA, USA; www.rstudio.com). Dplyr was used for data manipulation, Plotly for interactive graph development and Shiny for development of a user input-dependent and responsive interface. Next, HyperText Markup Language (HTML) and Cascading Style Sheets (CSS) were used to create a web layout. Finally, we hosted a Linux server with additional installation of the RStudio Shiny Server application. A web server was configured to make the application freely available to the public.

## Results

**Fig. 1:**
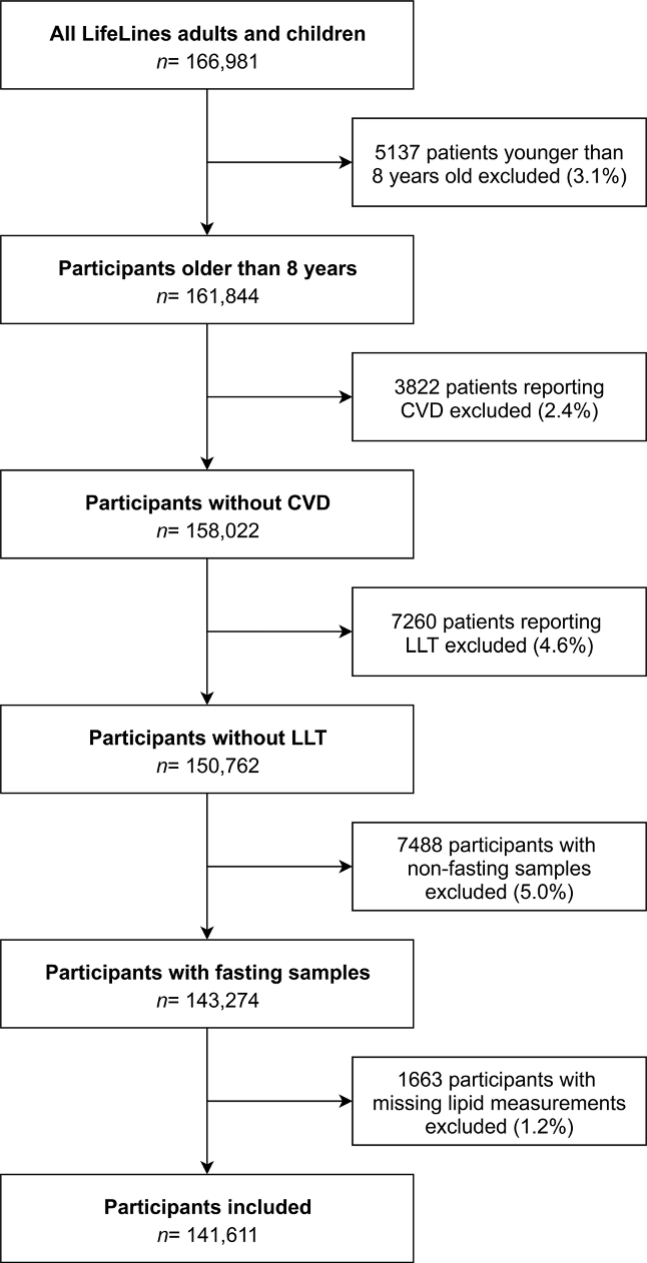
Flowchart of patient inclusion for lipid percentiles. Participants younger than 8 years old, reporting cardiovascular disease (*CVD*), reporting use of lipid-lowering therapy (*LLT*), with non-fasting samples or with missing lipid measurements were excluded

**Fig. 2:**
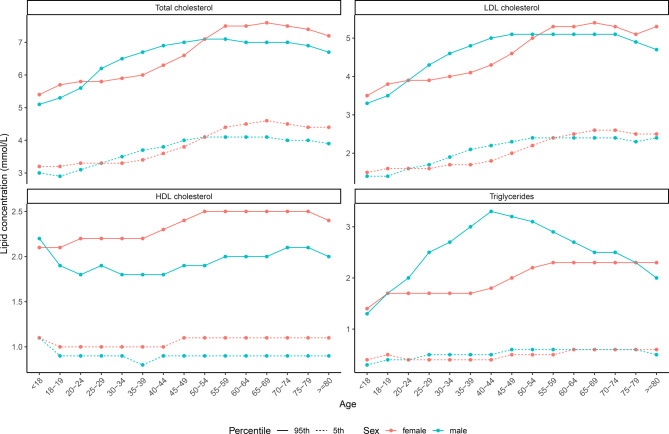
Fifth and ninety-fifth percentiles for total cholesterol, LDL cholesterol, HDL cholesterol and triglycerides (in mmol/L) per sex (male/female) and per age group (x-axis)

Of the 166,981 adults and children in the LifeLines cohort, 5137 participants (3.1%) younger than 8 years old were excluded (Fig. 1). In addition, the following participants were excluded: 3822 (2.4%) who had a CVD history, 7260 (4.6%) who were on lipid-lowering therapy, 7488 (5.0%) due to non-fasting samples and 1663 (1.2%) due to missing lipid measurements. After exclusion of a total of 25,370 participants, 141,611 participants (133,540 adults and 8071 children) were included in the current study. Numbers of included participants per age group are presented in Tab. 1. The 5th and 95th percentiles for total cholesterol, LDL cholesterol, HDL cholesterol and triglycerides per age and sex group are shown in Fig. 2.

**Table 1:** Number of included participants per age group, divided by sex

Age, years	Males	Females	Total
<18	3823	4248	8071
18–19	842	1616	2458
20–24	1905	3995	5900
25–29	4638	6576	11,214
30–34	5302	7059	12,361
35–39	6485	9535	16,020
40–44	8822	13,129	21,951
45–49	10,049	14,789	24,838
50–54	5705	8289	13,994
55–59	3338	5166	8504
60–64	3227	4502	7729
65–69	2182	2794	4976
70–74	1013	1284	2297
75–79	383	520	903
≥80	174	221	395
*Total*	*57,888*	*83,723*	*141,611*

Following the calculation of percentiles per group, we created input fields for total cholesterol, LDL cholesterol, HDL cholesterol, triglycerides, age, sex and unit of cholesterol measurements (i.e. mmol/L or mg/dL). After a user has filled in these fields, the output is formatted in a table displaying the plasma concentrations and corresponding percentiles. Simultaneously, output is displayed in an interactive graph for each plasma lipid feature separately. We ensured that the output instantly adapts to changes in the input boxes.

**Fig. 3:**
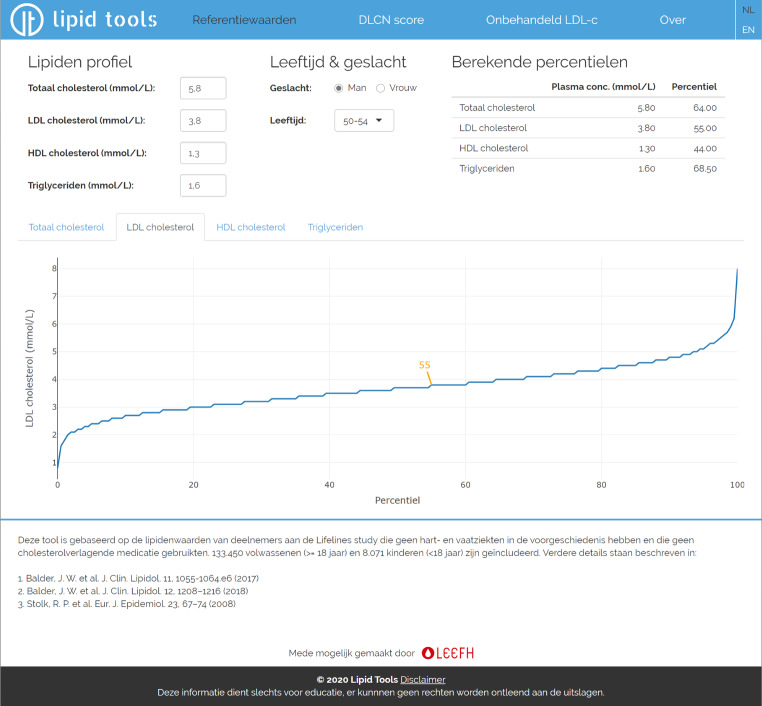
Screenshot from lipid reference tool in which obtained lipid percentiles are made publicly available. The tool consists of multiple input fields (i.e. lipid levels, age and sex) and multiple output formats. In the top right table, the lipid value and corresponding age- and sex-specific percentile are displayed. In the bottom half, a lipid trait-specific percentile graph is shown with a marker indicating the percentile based on the entered lipid values. The user can select a different lipid percentile graph using the tabs on top of the graph

Using an HTML template with CSS styling, we created a clear and simple responsive layout suitable for mobile and web use. After installing Shiny Server, we made the application available in both Dutch and English at www.lipidtools.com (Fig. 3), via the Dutch Foundation for Familial Hypercholesterolemia cascade screening programme (*Stichting Landelijk Expertisecentrum Erfelijkheidsonderzoek Familiaire Hart- en Vaatziekten*; www.leefh.nl) and at www.jojogenetics.nl.

## Discussion

We provided the first searchable, national lipid reference values in the Netherlands, which can be used in clinical cardiovascular practice, particularly in primary prevention. Our study showed that data collected in large-cohort studies can be made easily accessible owing to rapid advances in digital tools that combine simple statistical methods with standard programming languages.

Up to recently, non-searchable, outdated cohort data collected in the 1980s in the USA were used to provide lipid percentiles to Dutch physicians [11]. However, demographics of the current Dutch population, such as lifestyle, ethnicity and genetic factors, are largely different than those of the American population four decades ago, leading to a demand for up-to-date nation-specific lipid percentiles.

The availability of reference lipid percentile values is particularly important for diagnosing dyslipidaemias, since lipid values in all lipid traits change over the course of a lifetime with different magnitudes for both sexes, making it nearly impossible to know them by heart or to capture them in one table [2, 3]. The best-known genetic dyslipidaemia, for which specific percentiles play a pivotal role in diagnosis, is familial hypercholesterolaemia. Knowing whether a patient’s LDL cholesterol is high for his or her respective age and sex can raise the suspicion of this disease. The 95th percentile for the age- and sex-corrected LDL cholesterol value of family members is also required when the Dutch Lipid Clinic Network criteria are used to make the diagnosis of familial hypercholesterolaemia [12, 13]. The use of up-to-date age- and sex-specific percentiles will support a more accurate diagnosis.

In addition to providing more accurate and up-to-date reference values and thereby helping in the diagnosis of dyslipidaemias, our tool can contribute to increasing physicians’ awareness of lipid abnormalities. Ultimately, this could contribute to reducing the evidence-practice gap in lipid-lowering therapy use in the Netherlands [4, 5].

Epidemiological cohort studies have been performed for decades and are nowadays containing ever-increasing numbers of included patients; for example, the UK Biobank comprises data from > 500,000 participants [14]. Although results from these studies often form the basis for a better understanding of health and disease, most underlying data and statistical models are not accessible for use in clinical practice. For example, patterns in blood markers (such as lipids) or regression models predicting disease are widely published, but they are not easily incorporated in clinical care.

In general, our study showed that data collected in large-cohort studies can be made easily accessible with a combination of current digital tools. For example, these models could be incorporated in electronic health records [15] or, with the addition of easy-to-understand information and explanation, be made available for use by patients themselves [7]. We foresee that continuous developments in the digital health arena will lead to an accelerated implementation of such research outcomes in clinical care.

### Limitations

Our study has several limitations. First, it has been established that there are ethnic differences in lipid levels in the Netherlands [16]. As the LifeLines cohort consists of habitants from the three Northern Dutch provinces, more than 98% of the study population is of Caucasian/ West-European descent [17], making it uncertain whether these reference values can be applied to other ethnic groups.

Second, the cohort did not include secondary prevention patients. However, the proportion of patients excluded based on their CVD history was small (2.4%), which could have resulted in only a very small effect on the obtained percentiles, especially in younger individuals. Furthermore, this exclusion does not affect use of the tool in primary prevention, and comparison with healthy individuals could also be of use in secondary prevention.

Last, self-reported data were used for the history of CVD or lipid-lowering drug use, which resulted in a small risk of data imprecision.

## Conclusion

We developed the first clinically useful web-based tool that provides easy searchable and interactively displayed Dutch lipid profile percentiles, which can educate physicians on the normality or abnormality of lipid levels in clinical practice, particularly in primary prevention. This is not only of particular interest for dyslipidaemia screening in the Netherlands, but we also hope to provide a small step towards reducing the evidence-practice gap, mainly in primary prevention. In general, this study illustrates that data collected in big-cohort studies can be made easily accessible with modern digital techniques and preludes the digital health revolution yet to come.
